# Comparative effectiveness and safety of biosimilars versus reference biologics in rheumatoid arthritis during treatment initiation: a systematic review of real-world evidence

**DOI:** 10.1007/s11096-025-01956-6

**Published:** 2025-06-25

**Authors:** Chin Hang Yiu, Grace Tsz Yan Yau, Zoi Hei Wong, Chen-yun Lin, Richard O. Day, Jacques Raubenheimer, Christine Y. Lu

**Affiliations:** 1 The University of Sydney School of Pharmacy, Camperdown, NSW Australia; 2 Kolling Institute, Faculty of Medicine and Health, The University of Sydney and the Northern Sydney Local Health District, Sydney, NSW Australia; 3 School of Medicine and Health, University of New South Wales, Sydney, NSW Australia; 4 Department of Clinical Pharmacology & Toxicology, St Vincent’s Hospital Sydney and St Vincent’s Clinical Campus, UNSW Medicine, Sydney, NSW Australia; 5 Department of Pharmacy, Royal North Shore Hospital, St Leonards, NSW Australia

**Keywords:** Biologics, Biosimilars, Real-world evidence, Rheumatoid arthritis

## Abstract

**Background:**

Effective management of rheumatoid arthritis (RA) often requires the use of biological disease-modifying antirheumatic drugs (bDMARDs). Biosimilar drugs (biosimilars), licensed pharmaceutical products that exhibit high similarity to their reference biological products (originators), have emerged as more affordable alternatives.

**Aim:**

To compare the real-world effectiveness and safety of biosimilars and originators of bDMARDs in the management of RA at treatment initiation.

**Method:**

A systematic literature search was conducted using PubMed, MEDLINE, Embase, Scopus, International Pharmaceutical Abstract and CINAHL from database inception to 18th April 2025. Observational studies utilising real-world data (e.g., electronic health records, biologics registries) that compared clinical outcomes between patients initiating treatment with either a biosimilar or an originator for RA were included. Quality assessment was conducted using the Newcastle–Ottawa Scale (NOS) and a narrative synthesis was conducted to summarise key findings.

**Results:**

A total of 13 retrospective cohort studies were included, providing data on 34,280 patients initiating treatment with bDMARDs for RA. Treatment retention was the most investigated effectiveness outcome (n = 11), and all studies found that biosimilars were associated with comparable retention profiles compared to originators. No significant differences were identified for other effectiveness outcomes (e.g., disease activity indices). For safety outcomes, adverse events (AEs) were documented in eight studies. However, seven of these studies were of poor quality in assessing safety outcomes due to inadequate control for confounding factors.

**Conclusion:**

In real-world settings, biosimilars generally demonstrate comparable effectiveness to originators. Future investigations are warranted to examine the comparative safety profiles of biosimilars and originators.

**Supplementary Information:**

The online version contains supplementary material available at 10.1007/s11096-025-01956-6.

## Impact statements

- This review synthesises real-world evidence demonstrating that treatment retention is comparable between biosimilars and originator biologics in patients initiating therapy for rheumatoid arthritis. These findings provide reassurance to rheumatologists about the long-term effectiveness of biosimilars.
- The observed comparability in real-world settings reinforces the value of biosimilars as cost-effective alternatives, supporting formulary decisions and national policies that promote biosimilar uptake and reduce healthcare expenditure without compromising quality of care.
- Increased confidence in biosimilars should lead to expanded access to biologic therapies, especially in resource-constrained healthcare systems, potentially reducing treatment delays and promoting greater equity in care for patients with rheumatoid arthritis.

## Introduction

Rheumatoid arthritis (RA) is a systemic autoimmune disease characterised by chronic joint inflammation, which can lead to irreversible bone erosions, disability, and reduced quality of life [1]. The global burden is substantial, with an estimated prevalence of 460 per 100,000 population [2]. Effective management often requires biological disease-modifying antirheumatic drugs (bDMARDs), which are well-supported by evidence for improving disease outcomes and alleviating symptoms [1, 3–5]. However, their high cost—up to $36,000 USD annually per patient—can limit access to treatment [6].

Biosimilar drugs (biosimilars) are licensed products that are highly similar to their reference biologics (originators), with no clinically significant differences, and have emerged as more affordable alternatives [7]. Regulatory bodies such as the United States Food and Drug Administration (USFDA) and the European Medicines Agency (EMA) have approved biosimilars for several bDMARDs (e.g., adalimumab, etanercept, infliximab, rituximab) for the treatment of RA [8–11]. Biosimilars offer the potential to improve treatment access and reduce healthcare costs [8, 12]. Accordingly, clinical guidelines from organisations like the American College of Rheumatology (ACR) and European Alliance of Associations for Rheumatology (EULAR) support their use in RA [3, 4]. However, regulatory approvals and consensus-based recommendations for biosimilars have predominantly relied on data from randomised controlled trials (RCTs) [3, 4, 9]. For example, the first biosimilar for RA (infliximab biosimilar CT-P13), was approved by the USFDA and EMA based on the 54-week PLANETRA trial [13]. While these trials show comparable efficacy and safety to originators [14, 15], their typical short duration limits assessment of long-term outcomes such as treatment persistence [16]. Furthermore, RCTs often include younger, healthier patients, making it difficult to extrapolate findings to the broader, more diverse population seen in routine clinical practice [16].

Real-world observational studies, reflecting routine clinical practice over longer durations, are essential for comparing long-term treatment outcomes [16, 17]. Real-world data (RWD) refers to data routinely collected in clinical settings, outside the controlled environment of RCTs [18], and is increasingly recognised as key to understanding the comparative effectiveness of biologics in RA [19, 20]. While several systematic reviews have synthesised real-world evidence on patients switching from originators to biosimilars [21–24], less attention has been given to those initiating treatment with a biosimilar at the outset of RA management. Patients who switched from an originator to a biosimilar may have reduced treatment confidence due to concerns about efficacy or safety, contributing to a nocebo effect that negatively affects outcomes [25]. In contrast, treatment-naïve patients may have fewer preconceived concerns, potentially improving acceptance and adherence. As treatment initiation is a critical point in RA care, more evidence is needed to guide clinical decision-making in this population.

### Aim

The primary objective of this systematic review was to compare the real-world effectiveness and safety of biosimilars versus originators in RA patients initiating bDMARD therapy, addressing a key evidence gap. By focusing on treatment-naïve patients or those starting a new regimen, this review aimed to provide clinically relevant insights to inform evidence-based decisions at treatment initiation.

## Method

This review was reported in accordance with the Preferred Reporting Items for Systematic Reviews and Meta-analyses (PRISMA) guidelines [26]. The protocol was prospectively registered on PROSPERO (CRD42024542176).

### Search strategy

Six electronic databases—PubMed, MEDLINE, Embase, Scopus, International Pharmaceutical Abstract and CINAHL—were searched from inception to 29th April 2024. The search was updated through 18th April 2025 during manuscript review, confirming no new eligible studies. Key search terms included rheumatoid arthritis, biosimilars, and RWD (e.g., claims data, medical records). No restrictions were applied to publication date or languages. The full search strategy is available in Supplementary File 1. Grey literature, conference proceedings, and preprint servers were excluded to focus on peer-reviewed studies and ensure consistent quality assessment.

### Inclusion and exclusion criteria

This systematic review included original observational studies, such as cohort and case–control designs, that used RWD to compare clinical outcomes between biosimilars and originators in RA. RCTs, review articles, non-English publications, and conference abstracts without full-text articles were excluded. Studies on other rheumatic conditions (e.g., ankylosing spondylitis) were eligible only if separate data for RA were reported. Eligible studies used RWD collected from electronic health records (EHRs), medical claims data, and/or biologics registries. The exposures of interest were biosimilars of bDMARDs, with originator biologics as the comparator. Only studies involving patients initiating biosimilar or originator therapy, with outcomes assessed during follow-up, were included. Studies focusing on patients who switched from an originator to a biosimilar, or those without a comparator group (i.e., all participants received a biosimilar), were excluded, as the review aimed to compare outcomes between biosimilars and originators in RA. No restrictions were placed on the type of bDMARDs (e.g., tumour necrosis factor alpha [TNF-α] inhibitor, CD-20 inhibitor), baseline patient characteristics, or disease severity.

### Study selection

After removing duplicates, studies were imported into Covidence (Veritas Health Innovation, Melbourne, Australia) [27]. Titles and abstracts were independently screened by at least two investigators (C.H.Y, G.T.Y.Y, Z.H.W, C.L), with full texts reviewed for eligibility if deemed relevant. Disagreements were resolved through discussion or, when necessary, by consultation with a senior investigator (C.Y.L).

### Data extraction

Two investigators (C.H.Y, G.T.Y.Y) independently extracted data from eligible studies. Discrepancies were resolved through consensus or consultation with a senior investigator (C.Y.L). Extracted data included study characteristics (author, year, country, sample size, data source, intervention and control groups, follow-up duration, prior bDMARD exposure), and key findings on clinical outcomes. Outcomes were stratified into effectiveness (e.g., disease activity scores, treatment retention) and safety outcomes (e.g., adverse events [AEs]) based on previous systematic reviews of biologics in RA [28, 29]. Treatment persistence or retention was considered an effectiveness outcome, serving as a surrogate for long-term disease control in chronic inflammatory arthritis [28, 30, 31]. Measures included time to discontinuation (i.e., duration between index date and last prescription) and/or retention rates (i.e., proportion of patients remaining on treatment at specified follow-up points).

### Data synthesis

Due to heterogeneity in study populations, designs, follow-up durations, outcome measures, biosimilar agents, and statistical methods, a meta-analysis was not feasible. A narrative synthesis was therefore conducted to summarise the findings.

### Quality assessment

As all included studies were non-randomised, non-interventional observational cohorts, two investigators (C.H.Y, G.T.Y.Y) independently assessed study quality using the Newcastle–Ottawa Scale (NOS) for cohort studies [32]. Although inter-rater reliability was not formally calculated, discrepancies were resolved through discussion, or, when necessary, by consultation with a senior investigator (C.Y.L) to ensure consistency. Studies were rated according to the Agency for Healthcare Research and Quality (AHRQ) standards as good, fair, or poor quality [32]. Effectiveness and safety outcomes were evaluated separately due to differences in methodological approaches for these outcomes.

## Results

**Fig. 1:**
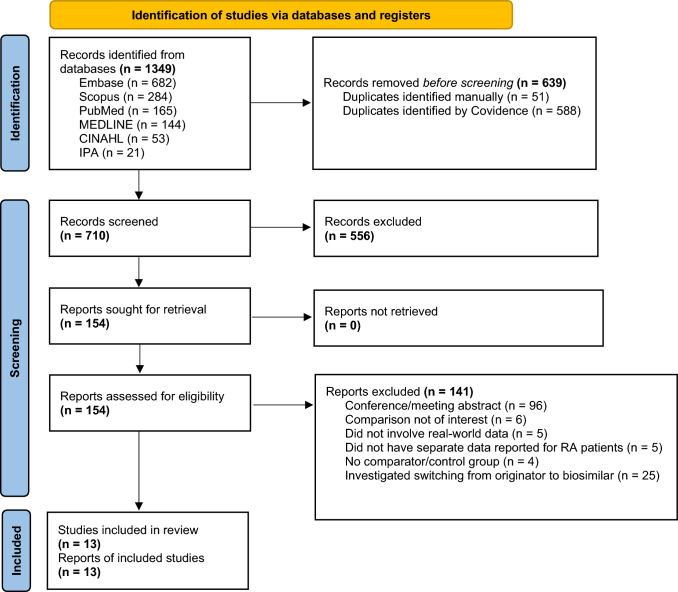
PRISMA flow diagram of study selection [26]. Abbreviations: IPA, International Pharmaceutical Abstracts; RA, rheumatoid arthritis

The electronic database searches generated 1,349 results. After removing duplicates, 710 articles remained. Following title and abstract screening, 154 full-text articles were assessed for eligibility based on inclusion and exclusion criteria. In total, 13 studies were included in this review (Fig. 1) [33–45].

### Study characteristics

Table 1 presents the characteristics of the included studies [33–45]. All were retrospective cohort studies conducted in either European countries (n = 10) [35–39, 41–45] or the Asia–Pacific region (n = 3) [33, 34, 40]. Collectively, the studies included 34,280 patients initiating treatment with a bDMARD for RA, with sample sizes ranging from 62 to 18,418 (median 441) patients [33–45]. Follow-up durations ranged from 3 months to 4 years [33–45].

**Table 1:** Baseline characteristics of all included studies (n = 13)

Author (year), country,	Data source	Intervention group(s), sample size (N)	Control group(s), sample size (N)	Observation/follow-up period	Inclusion of participants with previous exposure to any bDMARD(s)	Main clinical outcome(s) investigated
Kawakami et al. (2024) [33], Japan	EMRs data at Kyoto University Hospital from the KURAMA cohort	ETN biosimilar (LBEC0101), N = 23	ETN originator, N = 39	One year	Yes	AEs, DAS28, treatment retention
Deakin et al. (2024) [34], Australia	Customised EMRs data collected during routine clinical care across 43 clinics in Australia	ETN biosimilar (SB4), N = 141	ETN originator (Enbrel), N = 209	At least three months	Unclear	Treatment persistence
Larid et al. (2022) [35], France	EMRs data from the multicenter RIC-France registry	ADA biosimilar (Amgevita/Hulio) + ETN biosimilar (Benepali), N = 505	ADA originator (Humira) + ETN originator (Enbrel), N = 340	ADA: two years ETN: four years	Yes	Treatment persistence
Carballo et al. (2022) [36], Spain	EMRs data from a tertiary university hospital within the Barcelona public health system	ETN biosimilar (GP2015), N = 25	ETN originator, N = 90	52 weeks	Yes	DAS28, treatment persistence
Kearsley-Fleet et al. (2023) [37], United Kingdom	British Society for Rheumatology Biologics Register for RA (BSRBR-RA)	ETN biosimilar (Benepali/Erelzi), N = 797	ETN originator, N = 1009	One year	No (biologic-naïve patients only)	AEs, DAS28, EULAR response, HAQ, treatment retention
Popescu et al. (2022) [38], Romania	Romanian Registry of Rheumatic Diseases (RRBR)	ADA biosimilar (FKB327/GP2017/MSB11022/SB5), N = 228	ADA originator, N = 213	Six months	Yes	AEs, remission rates (Boolean, CDAI, DAS28, SDAI)
Pinto et al. (2022) [39], Portugal	EMRs data from the Rheumatic Diseases Portuguese Registry	ETN biosimilar (Benepali), N = 219	ETN originator (Enbrel), N = 645	36 months	No (biologic-naïve patients only)	AEs, ACR response, DAS28, EULAR response, treatment persistence
Sung et al. (2017) [40], Korea	BIOlogics Pharmacoepidemiology StudY (BIOPSY) of South Korea	INF biosimilar, N = 55	INF originator, N = 45	Four years	Yes	AEs, remission rates (ACR/EULAR Boolean index, CDAI, DAS28), treatment retention
Yazici et al. (2018) [41], Turkey	The Turkish Ministry of Health database which comprised from pharmacy, inpatient, outpatient, and laboratory claims	INF biosimilar (CT-P13), N = 204	INF originator, N = 575	> 12 months	Yes	Treatment retention
Jourdain et al. (2024) [42], France	French National Health Data System (SNDS)	ADA biosimilar (ABP501/MSB11022/ FKB327/GP2017/SB5) + ETN biosimilar (SB4/GP 2015) + INF (CT-P13/SB2), N = 10,699	ADA originator + ETN originator + INF originator, N = 7,719	One year	Yes	Treatment persistence
Codreanu et al. (2019) [43], Romania	Romanian Registry of Rheumatic Diseases (RRBR)	ETN biosimilar (SB4), N = 119	ETN originator, N = 123	Six months	Yes	AEs, DAS28
Di Giuseppe et al. (2021) [44], Sweden	Swedish Rheumatology Quality Register (SRQ)	ADA biosimilar (Amgevita/Hyrimoz/Idacio) + ETN biosimilar (Benepali/Erelzi) + INF biosimilar (Remsima/Inflectra/Flixabi/ Zessly) + RUT biosimilar (Ritemvia/Truxima/Rixathon), N = 5,483	ADA originator (Humira) + ETN originator (Enbrel) + INF originator (Remicade) + RUT originator (Mabthera), N = 3,880	One year	No (first-ever users only)	Treatment retention
Haugeberg et al. (2023) [45], Norway	EMRs data from five hospitals collected routine clinical practice	ETN biosimilar (SB4), N = 299	ETN originator, N = 575	Two years	Yes	AEs, DAS28, treatment persistence

ACR, American College of Rheumatology; ADA, adalimumab; AEs, adverse events; bDMARDs, biological disease-modifying antirheumatic drugs; CDAI, Clinical Disease Activity Index**;** DAS28, Disease Activity Score-28; EMRs, electronic medical records; ETN, etanercept; EULAR, European Alliance of Associations for Rheumatology; HAQ, Health Assessment Questionnaire; INF, infliximab; RA, rheumatoid arthritis; RUT, rituximab; SDAI, Simple Disease Activity Index

All included studies focused on patients initiating a new treatment episode with either a biologic or biosimilar [33–45]. Three studies included only biologic-naïve patients [37, 39, 44], while nine included patients with prior bDMARDs use. [33, 35, 36, 38, 40–43, 45]. One study did not report prior biologic exposure [34]. All studies investigated TNF-α inhibitors [33–45], most commonly etanercept (n = 9) [33, 34, 36, 37, 39, 42–45], followed by infliximab (n = 4) [40–42, 44], and adalimumab (n = 3) [38, 42, 44]. One study combined the results for adalimumab and etanercept [35]. Rituximab (CD-20 inhibitor) was investigated in only one study [44].

### Quality assessment

The quality assessment for effectiveness outcomes is summarised in Supplementary File 2. Seven studies were rated as good quality, exhibiting strong cohort comparability with adjustment for clinically relevant variables or use of propensity-score matching [34, 36, 37, 39, 42, 44, 45]. Six were rated poor due to lack of confounder adjustment when comparing biosimilars with originators [33, 35, 38, 40, 41, 43]. For safety outcomes, AEs were documented in eight studies (Supplementary File 3) [33, 36–40, 43, 45]. Most (n = 7) were rated poor quality due to reliance on descriptive statistics without confounder adjustment [33, 36–40, 43]. Only one study was rated good quality for using propensity-score matching [45].

### Effectiveness outcomes

Treatment retention was the most commonly investigated effectiveness outcome (n = 11) [33–37, 39–42, 44, 45]. Most studies examined retention rates over one to 3 years [33, 37, 39–42, 44, 45], while five investigated mean or median time to treatment discontinuation [34–37, 41]. Discontinuation of a bDMARD treatment episode was defined by a 60–90 day treatment gap in six studies [36, 39–42, 44], physician report in one study [37], and was not clearly defined in four studies [33–35, 45].

All studies found that biosimilars had comparable retention profiles to originators in real-world settings (Table 2) [33–37, 39–42, 44, 45]. Among studies on etanercept [33, 34, 36, 37, 39, 42, 44, 45], six found no significant difference in treatment retention between biosimilars and originators [33, 34, 36, 37, 39, 44], reporting comparable retention at 1 year [33, 36, 37, 44], persistence at 3 years [39], and median treatment duration (22.4 months vs 19.4 months, *p* = 0.95) [34]. However, two studies found better retention with the etanercept biosimilar [42, 45]. Notably, a large-scale study by Jourdain et al. (n = 11,265) found higher persistence with the biosimilar at 1 year (adjusted hazards ratio [HR] for discontinuation: 0.85, 95% confidence interval [CI] 0.78–0.93) [42]. Similarly, Haugeberg et al. (n = 1,455) observed higher retention rates for the biosimilar at weeks 52 (68% vs 52%) and 104 (60% vs 37%) in a propensity-score matched sample [45].

**Table 2:** Extracted main clinical outcomes in patients who initiated biosimilar versus originator for RA

Author (year)	Effectiveness outcome(s)	Safety outcome(s)
*Adalimumab* (n = 3)		
Popescu et al. (2022) [38]	*Boolean remission rate at six months:* 12.3% (B) versus 15.0% (O), *p* = 0.401 *CDAI remission rate at six months:* 12.3% (B) versus 15.5% (O), *p* = 0.329 *DAS28-CRP remission rate at six months:* 34.2% (B) versus 32.4% (O), *p* = 0.686 *DAS28-ESR remission rate at six months:* 15.8% (B) versus 21.1% (O), *p* = 0.148 *SDAI remission rate at six months:* 14.0% (B) versus 16.4% (O), *p* = 0.483	*Number of AEs at six months:* 8.7% (B) versus 40.8% (O) 51.4% of all recorded AEs were due to infection
Jourdain et al. (2024) [42]	*Treatment non-retention rate at one year:* 47% (B) versus 51% (O), adjusted HR 0.99 (95% CI 0.87–1.13), *p* = 0.881	NR
Di Giuseppe et al. (2021) [44]	*Treatment retention rate at one year:* HR for discontinuation was 0.93 (95% CI 0.73–1.18) for Imraldi (B); 1.32 (95% CI 1.0–1.72) for Amgevita (B); 0.86 (95% CI 0.62–1.19) for Hyrimoz(B), compared to Humira (O)	NR
*Adalimumab/etanercept combined* (n = 1)		
Larid et al. (2022) [35]	*Median treatment retention length:* Not calculable as did not fall under 50% (B) versus 23 months (O), *p* = 0.041	NR
*Etanercept* (n = 9)		
Kawakami et al. (2024) [33]	*Treatment retention rate at one year:* 74.4% (B) versus 58.7% (O), *p* = 0.06 *ΔDAS28-ESR score at 24 weeks:* − 2.75 (B) versus − 2.35 (O), *p* = 0.33	*Rate of AEs at one year:* 4.3% (B) versus 17.9% (O)
Deakin et al. (2024) [34]	*Median time to treatment discontinuation:* 22.4 months (B) versus 19.4 months (O), *p* = 0.95	NR
Carballo et al. (2022) [36]	*Mean time of treatment:* 46.51 (B) versus 46.74 weeks (O), *p* = 0.686 *ΔDAS28-CRP score at 52 weeks:* − 2.84 (B) versus − 2.37 (O), *p* = 0.372 Adjusted mean difference − 0.37 (95% CI − 1.03 to 0.29) *DAS28-CRP remission rate at 52 weeks:* 66.7% versus 54.6%, *p* = 0.303 *DAS28-ESR score at 52 weeks:* 2.6 (B) versus 2.1 (O), *p* = 0.377	*Rate of AEs at 52 weeks:* 4.0% (B) versus 7.8% (O)
Kearsley-Fleet et al. (2023) [37]	*Treatment retention rate at one year:* Adjusted HR for discontinuation = 1.15 (95% CI 0.99–1.33) Median time to discontinuation: 10 months (B) versus 11 months (O) Kaplan–Meier drug survival (one-year): 76% versus 71% *DAS28 remission at one year:* aOR = 0.88 (95% CI 0.70–1.11) *Good EULAR response at one year:* aOR = 0.86 (95% CI 0.69–1.07) *MCID in HAQ at one year:* aOR = 0.97 (0.73–1.28)	*Rate of AEs at one year:* 14% (B) versus 22% (O)
Pinto et al. (2022) [39]	*Treatment persistence rate at 36 months:* 72.6% (B) versus 63.6% (O), mean TOD 28.3 months (B) versus 27.4 months (O), *p* = 0.566 *ΔDAS score at 24 months:* − 2.3 (B) versus − 2.3 (O), *p* = 0.44 *Good EULAR response rate at 24 months:* 51.6% (B) versus 52.7% (O), *p* = 0.38 *ACR response* > *50% rate at 24 months:* 75% (B) versus 69.8% (O), *p* = 0.72	*Rate of AEs at 36 months:* 5.5% (B) versus 9.1% (O), *p* = 0.09
Jourdain et al. (2024) [42]	*Treatment non-retention rate at one year:* 42% (B) versus 48% (O), adjusted HR 0.85 (95% CI 0.78–0.93), *p* < 0.001	
Codreanu et al. (2019) [43]	*DAS28-CRP score at six months:* 3.3 versus 3.3, *p* = 0.829 *DAS28-ESR score at six months:* 3.9 (B) versus 3.8 (O), *p* = 0.785 Mean ΔDAS-ESR = − 2.5 (B) versus − 2.6 (O)	*Rate of AEs at six months:* 10.1% (B) versus 8.9% (O) 39.1% of all recorded AEs were due to infection
Di Giuseppe et al. (2021) [44]	*Treatment retention rate at one year:* HR for discontinuation was 0.92 for (95% CI 0.81–0.99) for Benepali (B), compared to Enbrel (O)	NR
Haugeberg et al. (2023) [45]	*Treatment persistence rate at 52 weeks:* 0.68 (B) versus 0.52 (O) *Treatment persistence rate at 104 weeks:* 0.60 (B) versus 0.37 (O) *DAS28 score at 52 weeks:* 3.2 (B) versus 3.0 (O) Mean difference in ΔDAS28 from baseline = − 0.02 (95% CI − 0.32 to 0.27)	*Rate of AEs at 104 weeks:* 14.0% (B) versus 19.2% (O) 3.2% of all recorded AEs were due to infection
*Infliximab* (n = 4)		
Sung et al. (2017) [40]	*Treatment retention rate at two years:* 52.6% (B) versus 36.9% (O), *p* = 0.98 *ACR/EULAR Boolean index remission rate at six months:* 15.0% (B) versus 7,1% (O) *p* = 0.46 *CDAI remission rate at six months:* 7.3% (B) versus 7.1% (O), *p* = 1.00 *DAS28-CRP remission rate at six months:* 35.0% (B) versus 42.9% (O), *p* = 0.69 *DAS28-ESR remission at six months:* 15.0% (B) versus 25.0% (O), *p* = 0.47	*Rate of AEs during observation period (four years):* 70.9% (B) versus 37.8% (O) 26.8% of all recorded AEs were due to infection
Yazici et al. (2018) [41]	*Treatment retention rate at 12 months:* 62.8% (B) versus 42.1% (O), mean time to discontinuation 288 days versus 177 days, *p* < 0.001	NR
Jourdain et al. (2024) [42]	*Treatment non-retention rate at one year:* 51% (B) versus 48% (O), adjusted HR 0.88 (95% CI 0.63–1.23), *p* = 0.300	NR
Di Giuseppe et al. (2021) [44]	*Treatment retention at one year:* HR for discontinuation was 1.14 (95% CI 0.92–1.41) for Flixabi (B), compared to Remicade (O)	NR
*Rituximab* (n = 1)		
Di Giuseppe et al. (2021) [44]	*Treatment retention at one year:* HR for discontinuation was 0.73 (95% CI 0.48–1.11) for Rixathon (B), compared to Mabthera (O)	NR

ACR, American College of Rheumatology; AEs, adverse events; aOR, adjusted odds ratio; B, biosimilar; CI, confidence interval; CDAI, Clinical Disease Activity Index**;** CRP, C-reactive protein**;** DAS28, Disease Activity Score-28; EMRs, electronic medical records; ESR, erythrocyte sedimentation rate; EULAR, European Alliance of Associations for Rheumatology; HAQ, Health Assessment Questionnaire; HR, hazards ratio; MCID, minimal clinically important difference; NR, not reported; O, originator; RUT, rituximab; SDAI, Simple Disease Activity Index; TOD, time-on-drug

For infliximab, three studies found no significant difference in 12-month retention [40, 42, 44], while one study (n = 779) reported higher retention with biosimilar CT-P13 (62.8% vs 42.1% at 12 months, *p* < 0.001) and longer persistence (288 days vs 177 days, *p* < 0.001) [41]. Adalimumab biosimilars also showed generally comparable retention [42, 44], though one small French study (n = 321) found longer median persistence with biosimilars (*p* = 0.041); however, this study combined data from adalimumab and etanercept users [35]. Rituximab biosimilar showed comparable retention in one study (HR for discontinuation: 0.73, 95% CI 0.48–1.11) [44].

Other effectiveness outcomes (e.g., Disease Activity Score-28 [DAS28], ACR/EULAR response rate, Boolean remission) were investigated in eight studies, with no significant differences observed between biosimilar and originator cohorts (Table 2) [33, 36–40, 43, 45]. DAS28 (i.e., change in DAS28 score and/or DAS28 remission rate) was the most commonly reported, assessed at 6 months [33, 38, 40, 43], 1 year [36, 37, 45], and 2 years [39].

### Safety outcomes

Among the eight studies reporting AEs [33, 36–40, 43, 45], six found lower AE rates in the biosimilar cohort (Table 2) [33, 36–39, 45]. Only Haugeberg et al. adjusted for confounders, showing lower AE rates with biosimilars (14.0% vs 19.2%) in a propensity-score matched sample [45]. One study reported similar AE rates (10.1% vs 8.9%) [43], while another found a higher rates in the biosimilar cohort (70.9% vs 37.8%) [40]. However, seven of the eight studies were rated as poor quality [33, 36–40, 43], limiting the strength of evidence on biosimilar safety in real-world settings. Notably, four studies identified infections (e.g., upper respiratory tract infections, pneumonia) as the most common AEs, with prevalence rates ranging from 3.2 to 51.4% [38, 40, 43, 45].

## Discussion

This is the first systematic review to synthesise real-world evidence from observational studies comparing the effectiveness and safety of biosimilars versus originators at treatment initiation for RA. Prior RCTs were limited by a median follow-up of 26 weeks and included only patients with prior methotrexate use [14]. In contrast, the observational studies in this review had longer follow-up durations (3 months to 4 years) and involved patients from routine clinical settings [33–45]. These studies also captured a diverse range of comorbidities (e.g., diabetes, cardiovascular diseases) and concurrent medications (e.g., conventional synthetic disease-modifying antirheumatic drugs [csDMARDs], corticosteroids) [33–45]. For instance, data on concomitant methotrexate use were available in 10 studies [33–41, 43–45]. However, no notable age differences were observed between participants in RCTs and those in the included studies. Our findings indicate that treatment persistence is the most frequently investigated longer-term effectiveness outcome and suggest biosimilars generally offer comparable retention profiles in real-world settings. While clinical recommendations based on RCTs support switching from originators to biosimilars in rheumatological diseases [46], some real-world studies have reported lower retention rates among switchers [23]. These studies often involved patients stable on bDMARD therapy [47, 48], whereas the studies in this review focused on patients initiating treatment [33–37, 39–42, 44, 45]. Individuals switching from an originator to a biosimilar may be more susceptible to the nocebo effect (i.e., negative expectations about a treatment), potentially reducing adherence [49, 50]. Therefore, treatment phase (e.g., initiation vs continuation) and patient perceptions should be considered when deciding to prescribe a biosimilar. Although four studies found potentially better retention with biosimilars [35, 40, 42, 45], two were rated as poor quality due to inadequate control for confounding [35, 40]. While one large, high-quality study showed higher retention for the etanercept biosimilar, no significant differences were observed for adalimumab or infliximab [42]. Overall, the available real-world evidence—though limited—suggests biosimilars and originators have comparable treatment persistence among patients initiating bDMARDs. Given previous concerns among rheumatologists about biosimilar efficacy [51, 52], our review provides important real-world evidence to help address these doubts and support informed clinical decision-making. Greater confidence in biosimilars may encourage broader uptake without compromising treatment outcomes, particularly where cost, policy, or access considerations are central.

This review also highlights limited real-world evidence on the comparable safety of biosimilars and originators. Although eight studies reported AE rates [33, 36–40, 43, 45], seven relied solely on descriptive statistics without adjusting for confounders [33, 36–40, 43]. While some suggested lower AE rates with biosimilars [33, 36–39], the clinical significance of these differences remain unclear due to lack of statistical adjustment. This highlights the need for future investigations using RWD and robust methods (e.g., propensity-score matching, multivariate regression) to reduce bias and better assess safety. Four studies identified infections as the most common AEs [38, 40, 43, 45]. Given the severity and high hospitalisation costs associated with infection-related AEs from bDMARDs [53, 54], more rigorous research is required to determine whether biosimilars carry a higher infection risk. Current real-world evidence remains insufficient to answer this question [38, 40, 43, 45].

**Fig. 2:**
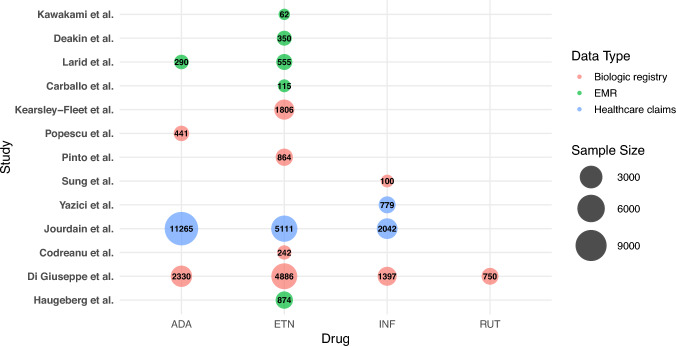
Diversity of real-world data among included studies. Abbreviations: ADA, adalimumab; EMR, electronic medical record; ETN, etanercept; INF, infliximab; RUT; rituximab

Our review also highlights key considerations for improving future real-world studies. Figure 2 summarises current data sources, biologics studied, and sample sizes. While 11 studies examined treatment persistence, only two used healthcare claims data—a more accurate and comprehensive source for capturing medication initiation and discontinuation than EMRs [55]. The underuse of claims data may limit the precision of treatment duration estimates. Future research should integrate claims data and clinical data to enable more robust analysis. In addition, all included studies focused on TNF-α inhibitors [33–45]. As biosimilars targeting other pathways become available [56], further research is warranted to evaluate their real-world effectiveness and safety. For instance, a tocilizumab (IL-6 inhibitor) biosimilar was recently approved by the EMA and USFDA for the treatment of RA, with a phase III equivalence trial demonstrating comparable efficacy, safety, and immunogenicity to its originator [57]. However, no observational studies comparing tocilizumab biosimilars with originators have been identified to date.

### Strengths and limitations

A key strength of this review is its comprehensive search strategy, which covered six electronic databases to identify relevant real-world studies. However, due to study heterogeneity, a meta-analysis was not feasible. While biosimilars are designed to be highly similar to their reference biologics, each has unique molecular characteristics and follows a distinct regulatory approval process. Therefore, generalising findings across all biosimilars should be done cautiously. Differences in manufacturing (e.g., post-translational modifications) can affect clinical outcomes, including immunogenicity [58]. Consequently, only a subset of biosimilars have received USFDA interchangeability designation, allowing pharmacy-level substitution without prescriber approval [59, 60]. It is important to distinguish between biosimilarity and interchangeability—these are separate regulatory concepts and not all biosimilars are considered interchangeable [59, 60]. These factors underscore the need to evaluate each biosimilar individually, particularly when interpreting real-world evidence across regulatory contexts. Additionally, most included studies were conducted in Europe [35–39, 41–45], which may limit generalisability to other regions. For example, our findings may not apply to lower-income settings, such as sub-Saharan Africa, where biosimilar uptake remains low [61]. Notably, no real-world studies from the United States or Canada were identified. In Canada, national and provincial non-medical switching policies have only recently been implemented [62, 63]. In the United States, biosimilar uptake has to date been limited due to regulatory, commercial, and reimbursement barriers [64]. However, recent developments—such as the USFDA’s proposal to eliminate switching study requirements for interchangeability and the use of more competitive pricing strategies—aim to facilitate greater biosimilar adoption [65]. Economic and policy-related factors—such as prescribing incentives, reimbursement structures, and out-of-pocket costs—may also affect biosimilar adoption but were not assessed in this review [66]. These factors could influence both uptake and outcomes and should be considered when interpreting findings across different health systems.

## Conclusion

In real-world settings, biosimilars generally show comparable effectiveness to originators. However, future investigations are warranted to clarify their safety profiles. Current evidence does not confirm full comparability between biosimilars and originators at treatment initiation for RA.

## Supplementary Information

Below is the link to the electronic supplementary material.

Supplementary file1 (DOCX 19 kb)

Supplementary file2 (DOCX 23 kb)

Supplementary file3 (DOCX 21 kb)
