# Supplementary material for: Comparative effectiveness and safety of biosimilars versus reference biologics in rheumatoid arthritis during treatment initiation: a systematic review of real-world evidence
Source: Int J Clin Pharm. 2025 Jun 25;47(6):1567–79. doi: 10.1007/s11096-025-01956-6 (PMC12630288; doi:10.1007/s11096-025-01956-6)
Supplement: Supplementary file 1 — Supplementary file1 (DOCX 19 kb) [file 11096_2025_1956_MOESM1_ESM.docx]

**Article title**:

Comparative effectiveness and safety of biosimilars and reference biologics in rheumatoid arthritis during treatment initiation: A systematic review of real-world studies

**Journal name:**

International Journal of Clinical Pharmacy

**Author names:**

Chin Hang Yiu (corresponding author),^1,2^ Grace Tsz Yan Yau,^1^ Zoi Hei Wong,^3^ Chen-yun Lin,^3^ Richard O. Day,^4^ Jacques Raubenheimer,^1^ Christine Y. Lu^1,2,5^

**Affiliations of the corresponding author:**

^1^ The University of Sydney School of Pharmacy, Camperdown, New South Wales, Australia

^2^ Kolling Institute, Faculty of Medicine and Health, The University of Sydney and the Northern Sydney Local Health District, Sydney, New South Wales, Australia

**Email address** **of the corresponding author:**

chin.yiu@sydney.edu.au

Supplementary File 1: Full search strategy

| **MEDLINE** | |
| --- | --- |
| 1 | arthritis, rheumatoid/ or arthritis, psoriatic/ or spondylarthritis/ |
| 2 | ("rheumatoid arthritis" or "psoriatic arthritis" or "ankylosing spondylitis").mp. |
| 3 | 1 or 2 |
| 4 | Biosimilar Pharmaceuticals/ |
| 5 | biosimilar*.mp. |
| 6 | 4 or 5 |
| 7 | Medical Records/ or Electronic Health Records |
| 8 | Databases, Pharmaceutical/ |
| 9 | ("claim*" or "administrative claim*" or "administrative data*" or "claim* data*" or "pharmacy data*" or "prescription data*" or "health* data*" or "health system*" or "medical record*" or medical chart* or "electronic health record*" or "real world").mp. |
| 10 | Comparative Effectiveness Research/ |
| 11 | 7 or 8 or 9 or 10 |
| 12 | 3 and 6 and 11 |
| **Embase** | |
| 1 | rheumatoid arthritis/ or psoriatic arthritis/ or spondylarthritis/ |
| 2 | ("rheumatoid arthritis" or "psoriatic arthritis" or "ankylosing spondylitis").mp. |
| 3 | 1 or 2 |
| 4 | biosimilar agent/ |
| 5 | biosimilar*.mp. |
| 6 | 4 or 5 |
| 7 | medical record/ or electronic health record/ |
| 8 | drug database/ |
| 9 | ("claim*" or "administrative claim*" or "administrative data*" or "claim* data*" or "pharmacy data*" or "prescription data*" or "health* data*" or "health system*" or "medical record*" or medical chart* or "electronic health record*" or "real world").mp. |
| 10 | comparative effectiveness/ |
| 11 | 7 or 8 or 9 or 10 |
| 12 | 3 and 6 and 11 |
| **International Pharmaceutical Abstract** | |
| 1 | ("rheumatoid arthritis" or "psoriatic arthritis" or "ankylosing spondylitis").mp. |
| 2 | biosimilar*.mp. |
| 3 | ("claim*" or "administrative claim*" or "administrative data*" or "claim* data*" or "pharmacy data*" or "prescription data*" or "health* data*" or "health system*" or "medical record*" or "medical chart*" or "electronic health record*" or "real world" or "comparative effectiveness").mp. |
| 4 | 1 and 2 and 3 |
| **Scopus** | |
| TITLE-ABS-KEY ( ( "rheumatoid arthritis" OR "psoriatic arthritis" OR "ankylosing spondylitis" ) AND ( biosimilar* ) AND ( "claim*" OR "administrative claim*" OR "administrative data*" OR "claim* data*" OR "pharmacy data*" OR "prescription data*" OR "health* data*" OR "health system*" OR "medical record*" OR "medical chart*" OR "electronic health record*" OR "real world" OR "comparative effectiveness" ) ) | |
| **CINAHL** | |
| (((MH "Arthritis, Rheumatoid") OR (MH "Arthritis, Psoriatic") OR (MH "Spondylarthritis")) OR ("rheumatoid arthritis" OR "psoriatic arthritis" OR "ankylosing spondylitis")) AND (((MH "Biosimilar Pharmaceuticals")) OR "Biosimilar*") AND (((MH "Electronic Health Records") OR (MH "Patient Record Systems") OR (MH "Medical Records") OR (MH "Medical Record Linkage")) OR ((MH "Databases, Health")) OR ((MH "Comparative Studies"))) | |
| **PubMed** | |
| ("rheumatoid arthritis" or "psoriatic arthritis" or "ankylosing spondylitis") AND (biosimilar*) AND ("claim*" or "administrative claim*" or "administrative data*" or "claim* data*" or "pharmacy data*" or "prescription data*" or "health* data*" or "health system*" or "medical record*" or "medical chart*" or "electronic health record*" or "real world" or "comparative effectiveness") | |
