# Supplementary material for: Comparative effectiveness and safety of biosimilars versus reference biologics in rheumatoid arthritis during treatment initiation: a systematic review of real-world evidence
Source: Int J Clin Pharm. 2025 Jun 25;47(6):1567–79. doi: 10.1007/s11096-025-01956-6 (PMC12630288; doi:10.1007/s11096-025-01956-6)
Supplement: Supplementary file 2 — Supplementary file2 (DOCX 23 kb) [file 11096_2025_1956_MOESM2_ESM.docx]

**Article title**:

Comparative effectiveness and safety of biosimilars and reference biologics in rheumatoid arthritis during treatment initiation: A systematic review of real-world studies

**Journal name:**

International Journal of Clinical Pharmacy

**Author names:**

Chin Hang Yiu (corresponding author),^1,2^ Grace Tsz Yan Yau,^1^ Zoi Hei Wong,^3^ Chen-yun Lin,^3^ Richard O. Day,^4^ Jacques Raubenheimer,^1^ Christine Y. Lu^1,2,5^

**Affiliations of the corresponding author:**

^1^ The University of Sydney School of Pharmacy, Camperdown, New South Wales, Australia

^2^ Kolling Institute, Faculty of Medicine and Health, The University of Sydney and the Northern Sydney Local Health District, Sydney, New South Wales, Australia

**Email address** **of the corresponding author:**

chin.yiu@sydney.edu.au

Supplementary File 2: Quality assessment for effectiveness outcome (n = 13 studies)

| **Author (Year)** | **Selection** | | | | **Comparability** | **Outcome** | | | **Quality**  **(AHRQ standards)** |
| --- | --- | --- | --- | --- | --- | --- | --- | --- | --- |
|  | Representativeness of exposed cohort  (Max: 1★) | Selection of the non-exposed cohort  (Max: 1★) | Ascertainment of exposure  (Max: 1★) | Demonstration that outcome of interest was not present at start of study  (Max: 1★) | Comparability of cohorts on the basis of design and analysis  (Max: 2★) | Assessment of outcome  (Max: 1★) | Was follow-up long enough for outcomes to occur^†^  (Max: 1★) | Adequacy of follow-up cohorts  (Max: 1★) |  |
| Kawakami et al. (2024) | ★ | ★ | ★ | ★ | –  No confounders were adjusted. Only included a statement that differences were not statistically significant between cohorts. | ★ | ★ | ★ | Poor |
| Deakin et al. (2024) | ★ | ★ | ★ | ★ | ★★  Propensity-score matching was performed to compare outcomes. Variables included age, gender, disease duration/activity, concomitant medication and comorbidities. | ★ | –  Did not specify follow-up period for participants. Only stated that patients needed to have at least three months of follow-up to be included in the study. | ★ | Good |
| Larid et al. (2022) | ★ | ★ | ★ | ★ | –  No confounders were adjusted. Only baseline characteristics between cohorts were reported in Table 1. | ★ | ★ | ★ | Poor |
| Carballo et al. (2022) | ★ | ★ | ★ | ★ | ★★  Analyses were adjusted for potential confounders including baseline characteristics (e.g., age, gender, comorbidities), concomitant medications (corticosteroids/csDMARDs). | ★ | ★ | ★ | Good |
| Kearsley-Fleet etal. (2023) | ★ | ★ | ★ | ★ | ★★  Propensity-score matched with variables including baseline characteristics (e.g., age, gender, BMI), disease activity (e.g., duration, activity scores), concomitant medications (csDMARDs). | ★ | ★ | ★ | Good |
| Popescu et al. (2022) | ★ | ★ | ★ | ★ | –  No confounders were adjusted. | ★ | –  Six months only. | ★ | Poor |
| Pinto et al. (2022) | ★ | ★ | ★ | ★ | ★★  Clinically relevant variables and all the variables with p-value < 0.20 from the univariate analysis were adjusted. | ★ | ★ | ★ | Good |
| Sung et al. (2017) | ★ | ★ | ★ | ★ | –  No confounders were adjusted. Only baseline characteristics between cohorts were reported in Table 1. Significant differences were observed in some variables (e.g., concomitant use of corticosteroids, DAS28-ESR scores). | ★ | ★ | ★ | Poor |
| Yazici et al. (2018) | ★ | ★ | ★ | ★ | –  No confounders were adjusted. Only baseline characteristics between cohorts were reported in Table 1. Significant differences were observed in some variables (e.g., age, comorbidities). P-values of most variables were also not reported (only stated as not significant). | ★ | ★ | ★ | Poor |
| Jourdain et al. (2024) | ★ | ★ | ★ | ★ | ★★  Propensity-score matched using variables including age, gender, comorbidities, drug history etc. | ★ | ★ | ★ | Good |
| Codreanu et al. (2019) | ★ | ★ | ★ | ★ | –  No confounders were adjusted. | ★ | –  Six months only. | ★ | Poor |
| Di Giuseppe et al. (2021) | ★ | ★ | ★ | ★ | ★★  Adjusted for age, sex, disease duration, concomitant use of csDMARDs and comorbidities. | ★ | ★ | ★ | Good |
| Haugeberg et al. (2023) | ★ | ★ | ★ | ★ | ★★  Confounders were adjusted age, sex, DAS28, order of biologics, and concomitant csDMARDs. | ★ | ★ | ★ | Good |

† Acceptable length of follow-up = one year

Good quality: 3 or 4 stars in selection domain AND 1 or 2 stars in comparability domain AND 2 or 3 stars in outcome/exposure domain

Fair quality: 2 stars in selection domain AND 1 or 2 stars in comparability domain AND 2 or 3 stars in outcome/exposure domain

Poor quality: 0 or 1 star in selection domain OR 0 stars in comparability domain OR 0 or 1 stars in outcome/exposure domain

Abbreviations: AHRQ, Agency for Healthcare Research and Quality; BMI, body mass index; csDMARDs, conventional synthetic disease-modifying antirheumatic drugs; DAS28, Disease Activity Score-28; ESR, erythrocyte sedimentation rate
