# Supplementary material for: Comparative effectiveness and safety of biosimilars versus reference biologics in rheumatoid arthritis during treatment initiation: a systematic review of real-world evidence
Source: Int J Clin Pharm. 2025 Jun 25;47(6):1567–79. doi: 10.1007/s11096-025-01956-6 (PMC12630288; doi:10.1007/s11096-025-01956-6)
Supplement: Supplementary file 3 — Supplementary file3 (DOCX 21 kb) [file 11096_2025_1956_MOESM3_ESM.docx]

**Article title**:

Comparative effectiveness and safety of biosimilars and reference biologics in rheumatoid arthritis during treatment initiation: A systematic review of real-world studies

**Journal name:**

International Journal of Clinical Pharmacy

**Author names:**

Chin Hang Yiu (corresponding author),^1,2^ Grace Tsz Yan Yau,^1^ Zoi Hei Wong,^3^ Chen-yun Lin,^3^ Richard O. Day,^4^ Jacques Raubenheimer,^1^ Christine Y. Lu^1,2,5^

**Affiliations of the corresponding author:**

^1^ The University of Sydney School of Pharmacy, Camperdown, New South Wales, Australia

^2^ Kolling Institute, Faculty of Medicine and Health, The University of Sydney and the Northern Sydney Local Health District, Sydney, New South Wales, Australia

**Email address** **of the corresponding author:**

chin.yiu@sydney.edu.au

Supplementary File 3: Quality assessment for safety outcomes (n = 8 studies)

| **Author (Year)** | **Selection** | | | | **Comparability** | **Outcome** | | | **Quality**  **(AHRQ standards)** |
| --- | --- | --- | --- | --- | --- | --- | --- | --- | --- |
|  | Representativeness of exposed cohort  (Max: 1★) | Selection of the non-exposed cohort  (Max: 1★) | Ascertainment of exposure  (Max: 1★) | Demonstration that outcome of interest was not present at start of study  (Max: 1★) | Comparability of cohorts on the basis of design and analysis  (Max: 2★) | Assessment of outcome  (Max: 1★) | Was follow-up long enough for outcomes to occur^†^  (Max: 1★) | Adequacy of follow-up cohorts  (Max: 1★) |  |
| Kawakami et al. (2024) | ★ | ★ | ★ | ★ | –  Descriptive statistics only. | ★ | ★ | ★ | Poor |
| Carballo et al. (2022) | ★ | ★ | ★ | ★ | –  Descriptive statistics only. | ★ | ★ | ★ | Poor |
| Kearsley-Fleet et al. (2023) | ★ | ★ | ★ | ★ | –  Descriptive statistics only. | –  Physician-reported. | ★ | ★ | Poor |
| Popescu et al. (2022) | ★ | ★ | ★ | ★ | ––  Descriptive statistics only. | ★ | ––  Six months only. | ★ | Poor |
| Pinto et al. (2022) | ★ | ★ | ★ | ★ | –  Descriptive statistics only. | ★ | ★ | ★ | Poor |
| Sung et al. (2017) | ★ | ★ | ★ | ★ | ––  Descriptive statistics only. | ★ | ★ | ★ | Poor |
| Codreanu et al. (2019) | ★ | ★ | ★ | ★ | ––  Descriptive statistics only. | ★ | ––  Six months only. | ★ | Poor |
| Haugeberg et al. (2023) | ★ | ★ | ★ | ★ | ★★  Propensity-score matching with variables such as age, sex, DAS28, order of biologics, and concomitant csDMARDs. | ★ | ★ | ★ | Good |

† Acceptable length of follow-up = one year

Good quality: 3 or 4 stars in selection domain AND 1 or 2 stars in comparability domain AND 2 or 3 stars in outcome/exposure domain

Fair quality: 2 stars in selection domain AND 1 or 2 stars in comparability domain AND 2 or 3 stars in outcome/exposure domain

Poor quality: 0 or 1 star in selection domain OR 0 stars in comparability domain OR 0 or 1 stars in outcome/exposure domain

Abbreviations: AHRQ, Agency for Healthcare Research and Quality; csDMARDs, conventional synthetic disease-modifying antirheumatic drugs; DAS28, Disease Activity Score-28
